# Phylogeny and Ontogeny of Erythropoiesis

**DOI:** 10.1155/2015/136270

**Published:** 2015-10-21

**Authors:** Wataru Nunomura, Aurora M. Cianciarullo, Takashi Kato, Ritsuko Shimizu, Malgorzata Witeska

**Affiliations:** ^1^Department of Life Science, Graduate School of Engineering and Resource Science, Akita University, Akita 010-8502, Japan; ^2^Research Center for Engineering Science, Graduate School of Engineering and Resource Science, Akita University, Akita 010-8502, Japan; ^3^Laboratory of Genetics, Butantan Institute, 05503-900 São Paulo, SP, Brazil; ^4^Program of Postgraduate Interunits in Biotechnology USP-IBU-IPT, University of São Paulo, 05508-900 São Paulo, SP, Brazil; ^5^Integrative Bioscience and Biomedical Engineering, Graduate School of Advanced Science and Engineering, Waseda University, Tokyo 162-8480, Japan; ^6^Department of Biology, Faculty of Education and Integrated Arts and Sciences, Waseda University, Tokyo 162-8480, Japan; ^7^Department of Molecular Hematology, Tohoku University Graduate School of Medicine, Sendai 980-8575, Japan; ^8^Department of Animal Physiology, University of Natural Sciences and Humanities, 08-110 Siedlce, Poland

Erythrocytes are essential for survival of vertebrates due to their ability to take up, bind, and transport oxygen. Recent study clarified that the number of cells in adult human is ~3.7 × 10^13^ cells and about 2/3 are erythrocytes (Bianconi et al. (2013)* Ann Hum Biol*. 40: 463). Although this fact indicates the importance of these cells, the following questions still remain: why only mammalian erythrocytes have no nucleus? And what is the mechanism that works in enucleation of mammalian erythroblasts? There is no doubt that we explore answers to these questions in the evolution of not only vertebrates but also their ancestors. We well understand that malaria infection and abnormal shape of erythrocytes are major critical diseases of human erythrocytes. The cause of diseases will be clarified by studies on the evolutional background of vertebrates. Based on the evolutional biological background, cellular, biochemical, and biophysical approach to these problems will lead us to the response. Standing on the concepts, this special issue is containing four reviews and two original papers, both of basic scientific and clinical approaches.

Frog is biologically very interesting animal. They perform metamorphosis and change habitat from aquatic to terrestrial during their life cycle. In the young period, they generally perform metamorphosis from larva with gills to an adult air-breathing form with lungs. Amphibian larvae, tadpoles, live in freshwaters and breathe with external gills, while the adults spend most time on land and use lungs. Amphibians also use their skin as a secondary respiratory surface but some of them lack lungs and rely entirely on their skin. The earliest amphibians evolved in the Devonian period from sarcopterygian fish with lungs and bony-limbed fins, features that were helpful in adapting to terrestrial life. In the field of erythropoiesis, it is very interesting that amphibian erythrocytes show much larger size than those of the other vertebrates. S. Maekawa and T. Kato presented the extensive review of the present understanding of erythropoiesis in vertebrates, focusing on erythropoietic responses to environmental stress and pointed out that diversity to the hematopoietic responses to environmental stress is still not clear. They showed the importance of regulation pathways of erythropoietin and hypoxia-inducible factor (HIF) in maturation of the erythrocytes in stress induced by hypoxia and low temperature.

Thrombocytes (platelets) participate in blood coagulation to stop bleeding in case of blood vessel injuries. As well as erythrocytes, mammalian platelets also do not have nucleus. They develop as fragments of cytoplasm that are derived from the megakaryocytes in the bone marrow and then enter the circulation. These inactive platelets are biconvex discoid (lens-shaped) structures of 1–4 *μ*m diameter. In the other vertebrates thrombocytes circulate as intact mononuclear cells. The origin of thrombocytes and erythrocytes in vertebrates and the regulation mechanism of differentiation of these cells from stem cells are extremely interesting. O. Svoboda and P. Bartunek reviewed and summarized the recent knowledge about the origin and development of erythrocytes and thrombocytes including regulatory factors and signal transduction. One of the most interesting issues in the hematopoiesis is how the erythroblasts exclude their nuclei and what biological significance of enucleation is.

Erythroblastic island is thought to be essential for maturation of erythroblasts and enucleation is mammalian specific event. The erythroblastic island consists of macrophage in its center and erythroblasts located around it. Macrophages play an important role in erythroblast proliferation and differentiation. This indicates that cell-cell interaction is essential for erythropoiesis. Macrophages also play an important role as scavengers of extruded nuclei to avoid producing autoantibodies. However, the details of biological significance of erythroblastic island are still to be elucidated. K. M. Giger and T. A. Kalfa reviewed and discussed the importance of the macrophages in erythroblast enucleation based on phylogenetic development of erythroblastic islands in mammals.

Heme is essential molecule for the transportation (hemoglobin) and storage (myoglobin) of oxygen. In contrast to the vertebrates in which hemoglobin in closed in the erythrocytes, the cells specialized in oxygen transport invertebrates show hemoglobin dissolved in hemolymph. Oxygen level in atmosphere increased due to photosynthesis by cyanobacteria and oxygen is essential for producing energy (ATP) in most living beings, and oxygen binding proteins evolved. The pathway of heme biosynthesis in erythroblasts is very complex. It takes place in cytoplasm and in mitochondria, with participation of numerous enzymes, for example, ALAS (5-aminolevulinic acid synthase). Although the enzymatic products (intermediate metabolites) are transported through the mitochondrial membrane, the mechanism and related molecule(s) are unknown. Mutations in* ALAS2* gene cause microcytic anemia and the appearance of ring sideroblasts in the bone marrow. T. Fujiwara and H. Harigae reviewed and discussed the biosynthesis, regulation and transporting system of heme in mammalian erythroid cells and the disorders of heme biosynthesis from the clinical point of view.

Malaria infection is very critical problem in the world. The World Health Organization reports that malaria infection is a life-threatening disease caused by parasites that are transmitted to people through the bites of infected mosquitoes. In 2013, 198 million cases of malaria disease worldwide caused an estimated 584,000 to 755,000 deaths (World Malaria Report, 2014), mostly among African children. The malaria parasites require invasion to the erythrocytes in its final stage of life cycle in human. The global warming caused spreading of the mosquito transmitting* Plasmodium falciparum* in the world. Although a number of studies of the malaria infection have been performed, there are few comparative studies regarding the biophysical properties of the infected erythrocytes. E. H. Hayakawa et al. reported their original data of the difference in* zeta* potential of malaria parasite infected erythrocytes in human and mouse. Based on the relationships between biophysical analysis and images of shape by scanning electron microscopy (SEM), the authors also discussed the species specificities of parasite infection to hosts.

Membrane skeleton contains heterotetramers of spectrin *αβ* that line the intracellular side of the membrane in erythrocytes and other eukaryotic cells. Spectrin is bound to anchoring proteins, protein 4.1R (80 kDa erythrocyte type) or ankyrin. Protein 4.1R also binds to short actin filament with spectrin in its middle region and to glycophorin C, transmembrane protein, and p55 in its N-terminal 30 kDa membrane binding domain, FERM (four-one, ezrin, radixin, moesin) domain. Ankyrin anchors spectrin network and band 3 (anion exchanger (AE) 1), that is, exchange of HCO^−^ in the cell and Cl^−^ outside of the cells. The biconcave shape of human erythrocytes is maintained by these protein interactions. The mutations and disorders of these proteins cause abnormal shape of the erythrocytes, for example, hereditary spherocytosis (HS), hereditary elliptocytosis (HE), and hereditary pyropoikilocytosis (HPP). The establishment of simple method of diagnosis of these erythrocyte anomalies facilitates the laboratory examination. Eosin-5-maleimide (EMA) is a fluorochrome that primarily binds to band 3. S. Suemori et al. investigated the relationship among decreasing of EMA binding to erythrocytes, electrogram of membrane skeletal proteins by SDS-PAGE, and their shape examined by SEM in 12 patients.

The disorders of erythrocytes are also thought as a “trade-off” of animal evolution, especially human. About ~2 × 10^11^ of erythrocytes per day are produced in adult human, which means that the same number of erythroblasts excludes their nuclei and converts to mature erythrocytes. By the insight of evolutionary biology, we should begin to elucidate the biological problems which are still unsolved, in order to advance to the field of the hematological diseases. We believe that evolutional biology will help to solve the problems of the origin and development of hematological diseases. We hope that this special issue will contribute to the modern knowledge of erythropoiesis in vertebrates.

